# Genetic variation among 481 diverse soybean accessions, inferred from genomic re-sequencing

**DOI:** 10.1038/s41597-021-00834-w

**Published:** 2021-02-08

**Authors:** Babu Valliyodan, Anne V. Brown, Juexin Wang, Gunvant Patil, Yang Liu, Paul I. Otyama, Rex T. Nelson, Tri Vuong, Qijian Song, Theresa A. Musket, Ruth Wagner, Pradeep Marri, Sam Reddy, Allen Sessions, Xiaolei Wu, David Grant, Philipp E. Bayer, Manish Roorkiwal, Rajeev K. Varshney, Xin Liu, David Edwards, Dong Xu, Trupti Joshi, Steven B. Cannon, Henry T. Nguyen

**Affiliations:** 1 Division of Plant Sciences and National Center for Soybean Biotechnology, University of Missouri, Columbia, MO 65211 USA; 2 Department of Agriculture and Environmental Sciences, Lincoln University, Jefferson City, MO 65101 USA; 3 USDA-ARS Corn Insects and Crop Genetics Research Unit, Ames, IA 50011 USA; 4 Department of Electrical Engineering and Computer Science, and Christopher S. Bond Life Sciences Center, University of Missouri, Columbia, MO 65211 USA; 5 Institute of Genomics for Crop Abiotic Stress Tolerance, Department of Plant and Soil Science, Texas Tech University, Lubbock, TX 79409 USA; 6 MU Institute of Data Science and Informatics, University of Missouri, Columbia, MO 65211 USA; 7 Department of Agronomy, Iowa State University, Ames, IA 50011 USA; 8 USDA-ARS, Soybean Genomics and Improvement Lab, Beltsville, MD 20705 USA; 9 Bayer CropScience, St. Louis, MO 63141 USA; 10 Corteva Agriscience, Indianapolis, IN 46268 USA; 11 Pairwise Plants LLC, Durham, NC 27709 USA; 12 Bayer CropScience, Research Triangle Park, NC 27709 USA; 13 School of Biological Sciences, The University of Western Australia, Perth, WA 6009 Australia; 14 International Crops Research Institute for the Semi-Arid Tropics (ICRISAT), Patancheru, Hyderabad, Telangana 502324 India; 15 Beijing Genomics Institute-Shenzhen, Shenzhen, 518083 China; 16 State Key Laboratory of Agricultural Genomics, China National GeneBank, BGI-Shenzhen, Shenzhen, 518083 China; 17 Department of Health Management and Informatics, University of Missouri, Columbia, MO 65211 USA

**Keywords:** Plant breeding, Plant sciences

## Abstract

We report characteristics of soybean genetic diversity and structure from the resequencing of 481 diverse soybean accessions, comprising 52 wild (*Glycine soja*) selections and 429 cultivated (*Glycine max*) varieties (landraces and elites). This data was used to identify 7.8 million SNPs, to predict SNP effects relative to genic regions, and to identify the genetic structure, relationships, and linkage disequilibrium. We found evidence of distinct, mostly independent selection of lineages by particular geographic location. Among cultivated varieties, we identified numerous highly conserved regions, suggesting selection during domestication. Comparisons of these accessions against the whole U.S. germplasm genotyped with the SoySNP50K iSelect BeadChip revealed that over 95% of the re-sequenced accessions have a high similarity to their SoySNP50K counterparts. Probable errors in seed source or genotype tracking were also identified in approximately 5% of the accessions.

## Abstract (summary)

**Table:** 

Measurement(s)	genetic variation • SNP • Linkage Disequilibrium
Technology Type(s)	DNA sequencing • bioinformatics analysis • computational phylogenetic analysis
Sample Characteristic - Organism	*Glycine soja* • *Glycine max*

Machine-accessible metadata file describing the reported data: 10.6084/m9.figshare.13568552

## Background & Summary

Soybean [*Glycine max* (L.) Merr.] is one of the major grain legumes and oil seeds cultivated worldwide, particularly in Asia and the Americas. The cultivated soybean, *G. max*, was domesticated from its wild relative, *G. soja*, around the Eleventh Century B.C, in Eastern China^1^. Cultivated soybean spread to other locations through Asia shortly following domestication, and was then introduced into the United States in 1765^1^. Soybean lost genetic diversity through domestication-related genetic bottlenecks, while the wild relative *G. soja*, growing in various environmental conditions, retained significant genetic diversity^2,3^.

The first reference genome for cultivated soybean was released in 2010^4^, followed by high-quality assemblies of several other soybean accessions, including elite lines and wild soybean accessions^5–10^. This genomic sequencing has been complemented by resequencing projects (summarized in Figshare file F1^11^), designed to assess genetic variation across broader samplings of soybean germplasm^3,5,12,13^.

To map patterns of genome-wide variation, population structure, and to facilitate identification of the genetic basis of agronomic traits in soybean, 481 accessions from the USDA Soybean Germplasm Collection were re-sequenced. This included modern cultivars, traditional landraces, and wild species accessions, from throughout the range of both domesticated and wild soybean (Figshare file F2^11^). All of the reads were mapped onto the Williams 82 reference genome assembly and approximately 7.8 million single nucleotide polymorphisms (SNPs) were identified from the mapped reads. The *G. max* Williams 82 assembly was used as reference because that is most widely used and accepted. We compare these results from the chip-based genotyping of the same genotypes from the SoySNP50K iSelect BeadChip^14^ results, helping validate both results, while also identifying several probable errors in germplasm tracking or labeling. The genotypic data was also used to evaluate genetic relationships and population structure. We provide the variant data and associated analyses as major resources for other researchers, toward better understanding of soybean diversity for continued genetic improvement of soybeans.

## Methods

### Germplasm selection for sequencing

The USDA Soybean Germplasm Collection contains 20,035 accessions, including 1,168 *G. soja* and 18,867 *G. max* lines. The SoySNP50K BeadChip^14^ was utilized for genotyping these accessions and for selecting a core set of diverse *G. max* (1,148) and *G. soja* (81) lines for further studies. The core set maintained 98.7% and 96.1% of the diversity of the entire cultivated and wild soybean collections, respectively^14^. To maximize diversity without losing genetic information, we selected a total of 481 diverse soybean accessions from the core mentioned above (429 of *G. max* and 52 of *G. soja*) for sequencing and analysis (Figshare file F2^11^). The germplasm of the 481 accessions comes from 37 countries, with the largest numbers coming from China (223), Japan (56), the U.S. (54), South Korea (44), the Russian Federation (26), and North Korea (14).

### Soybean sample collection, DNA sequencing, and read-mapping

Soybean seeds were obtained from the Soybean Stock Center (USDA-GRIN). For each accession 50 seeds were planted, in a greenhouse at the University of Missouri. After two weeks (V1 growth stage) a minimum of 24 plants per accession were selected based on morphological homogeneity (hypocotyl color, plant height, leaf size) for leaf sample collection. Leaf samples were flash frozen and high quality high molecular weight DNA extractions were conducted using a standard protocol^15^. Soybean samples were tested for the heterogeneity using the SoySNP6K Illumina Infinium BeadChips (Illumina, Inc. San Diego, CA)^16^ and the samples with below 10% heterogeneity were selected for whole-genome sequencing. A total of 5 µg of genomic DNA from each soybean line was used to construct the sequencing library, following the Illumina sequencing protocols. Paired-end sequencing libraries with an insert size of ~300 bp were sequenced using an Illumina HiSeq. 2000 sequencer at the Beijing Genomics Institute (BGI). The 50 most diverse lines from the core set were sequenced at a 40x genome equivalent and the remaining samples were sequenced at 15x coverage (Figshare file F3^11^). This generated a total of 7.5 TB of raw next generation sequencing (NGS) reads data from all samples.

The reference genome for the soybean cultivar Williams 82^4^ (Wm82.a2) (a widely used reference assembly), downloaded from Phytozome (www.phytozome.net)^17^, was used for mapping. We built the PGen^18^ multi-step SNP identification workflow using the Pegasus^19^ workflow management system (Pegasus-WMS), for quality checks, alignment of reads, variants calling, variants filtration, and VCF merging. The workflow takes paired-end and single-end Fastq reads as input, and performs data quality checks using FastQC^20^. Filtered, high-quality reads are then aligned against the reference genome using BWA^21^. Picard Tools^22^ is also used at this step to locate duplicate molecules and assign all reads into groups (using default parameters). The bwa command is ‘bwa mem -t 12 -M ref.fastq paired_read1.fastq paired_read2.fastq > aln.sam’.

### Variant detection

After the sequence alignment, SNPs and indels were called using the Haplotype caller algorithm from the Genome Analysis Toolkit (GATK)^23^ version gatk-2.5-2-gf57256b. Filtering criteria are provided in the INFO fields in the VCF file. Important abbreviations in this section are: QD, quality by depth; FS, Fisher strand values; and MQ, mapping quality of variants. Detected variants were then filtered using the criteria “QD < 26.0 || FS > 60.0 || MQ < 40.0” for SNPs and “QD < 26.0 || FS > 200.0 || MQ < 40.0” for indels. Additional filtering can also be applied by modifying the configuration file of a PGen workflow. Outputs were generated as BAM and VCF standard formats that were stored in the CyVerse data store^24^ and accessed via the SoyKB database^25^ via the NGS re-sequencing data browser^25,26^, and for download at the SoyBase Data Store^27^. SNPs were assigned IDs using the script “assign_name.awk” available at https://github.com/soybase/SoySNP-Names. Missing data accounted for 1% and heterozygous SNPs accounted for 0.5% of the total dataset (Figshare file F4^11^). SNPs were annotated using SnpEff 3.0^28^ (Figshare file F5^11^). In SoyKB the data is also loaded into the SNPViz 2.0 tool^29^ for interactive exploration of accession relationships using SNPs in selected genomic regions.

### Phylogenetic analysis

**Fig. 1:**
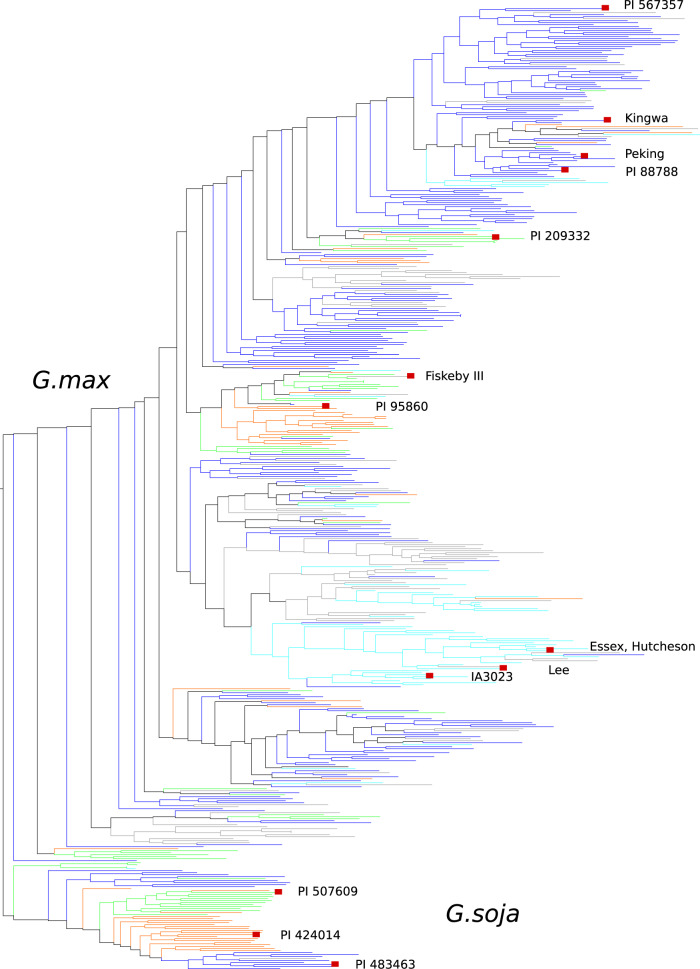
Phylogenetic tree of the 481 re-sequenced accessions. The tree is rooted between *G. max* and *G. soja* accessions. Colors indicate countries of origin: blue, China; orange, Korea; green, Japan; cyan, United States; gray, all others (predominantly from Russia). Cultivars of interest are highlighted on the tree.

25,496 SNPs in common with the SoySNP50K iSelect BeadChip-derived data were used to develop the phylogenetic tree (Fig. 1 and Data File glyma.Wm82.gnm2.div.G787.sampled_25Kpos.tree, SoyBase Data Store^30^). To generate an alignment suitable for phylogenetic reconstruction, every 5^th^ SNP was selected, giving an alignment length of 4,518 characters (Data File glyma.Wm82.gnm2.div.G787.sampled_25Kpos.fna, SoyBase Data Store^30^). This moderate matrix size permits maximum likelihood phylogenetic reconstruction and also takes advantage of the SNP distribution in the SoySNP50K set, as the SoySNP50K SNPs were chosen relative to LD, with greater density at chromosome ends and less density in the high-LD pericentromere, and avoiding closely-spaced SNPs. A tag was added to the genotype identifiers to indicate country of origin (Figshare file F2^11^). A maximum likelihood phylogenetic tree was calculated using FastTree^31^, version 2.1.8, with default nucleotide parameters. Tree visualizations were generated using the Archaeopteryx tree viewer^32^.

### Linkage disequilibrium (LD) analysis

The extent and rate of LD decay was calculated as the square of the correlation coefficient (*r*^*2*^) between pairwise comparisons of all SNPs across a chromosome (Table 1, Supplementary Figure 1). For LD analysis accessions were divided into two groups: *G. max* and *G. soja*, to compare the LD between wild and cultivated soybeans. Pairwise *r*^*2*^ measures were calculated using PLINK v 1.90b4.4^33^ using parameters *-- dog --r2 --ld -window-r2 0 --ld-window 999999 --ld-window-kb 80000*. For each chromosome, *r*^*2*^ values were plotted against inter-marker distances to visualize rate of decay at the baseline level where *r*^*2*^ = 0.2. The decay curve was estimated using the Hill and Weir formula^34^.

**Table 1:** Average decay of LD (*r*^*2*^) as a function of physical distance between pairs of loci across soybean chromosomes.

Chromosome	Chromosome size (Mb)	LD decay distance (Mb)
Gm20	47.88	0.061
Gm19	50.68	0.199
Gm18	57.97	0.292
Gm17	41.62	0.093
Gm16	37.83	0.053
Gm15	51.67	0.192
Gm14	48.98	0.098
Gm13	45.81	0.080
Gm12	40.01	0.084
Gm11	34.73	0.105
Gm10	51.50	0.092
Gm09	50.15	0.118
Gm08	47.80	0.160
Gm07	44.61	0.276
Gm06	51.32	0.081
Gm05	42.19	0.139
Gm04	52.37	0.077
Gm03	45.70	0.065
Gm02	48.57	0.097
Gm01	56.83	0.105
Average	47.41	0.123
Correlation		0.33

Pairwise comparisons are shown for the first 1000 kb distance. Decay curves are plotted following the Hill and Weir method (Supplementary Figure 1). The curve in green represents average decay for all accessions whereas the blue and red curves represent average decay in G. *soja* and G. *max* accessions, respectively.

### Structure analysis

**Fig. 2:**
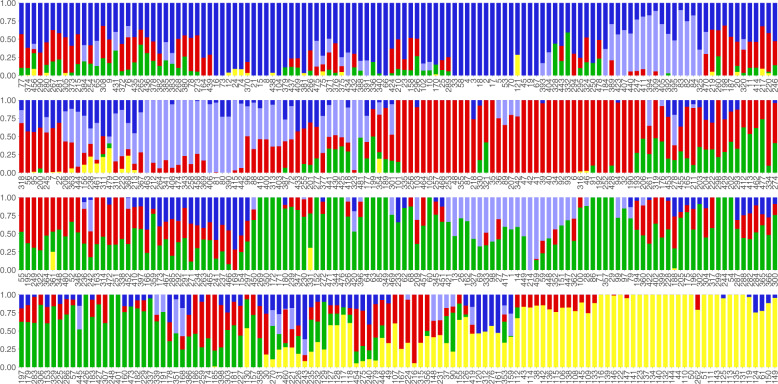
Genetic Structure plot for the 481 re-sequenced accessions. Accessions are plotted in the same order as in the phylogenetic tree in Fig. 1. Numbers indicated below each structure represent the position of that accession in the VCF file. The plot was constructed using cluster size of K = 5. Accessions in yellow generally correspond with *G. soja*.

Structure within the collection was calculated using the Bayesian clustering program FastStructure^35^ using a logistic prior for K ranging 1 to 10. The script chooseK.py (part of the FastStructure distribution) was used to determine the best K that explained the structure in the collection based on model complexity. Structure was visualized (Fig. 2) using an R package Pophelper v2.3.0^36^.

**Fig. 3:**
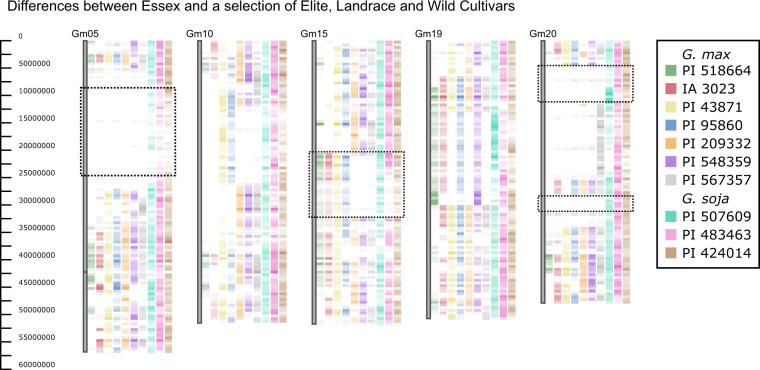
Heatmap comparison of cultivated and wild accessions to elite breeding line Essex. The darker the color, the more differences between the given accession and Essex. White regions indicate no differences between the given accession and Essex (PI 548667). Accessions are ordered and color-coded from left to right as follows: green, PI518664 (Hutcheson); red, IA3023; yellow, PI438471 (Fiskeby III); blue, PI 95860 (*G. max* line from Korea); orange, PI 209332 (*G. max* line from Japan); purple, PI 548359 (Kingwa); gray, PI 567357 (top of phylogenetic tree); turquoise, PI 507609 (*G. soja* from Japan); pink, PI 483463 (*G. soja* from China); brown, PI 424014 (*G. soja* from Korea).

Chromosomal similarity heatmaps displayed in Fig. 3 were created using the tool GCViT (Genotype Comparison Visualization Tool^37,38^), available for interactive use at https://soybase.org/gcvit. Soybean accession Essex (PI 548667) was selected to be the reference genotype due to its importance in soybean breeding programs. Bin size is set to 500,000 with the right side of the chromosomes set to display type “heatmap” showing SNP differences between the selected accessions and Essex. Heatmap base color set to white going from min-max.

### Comparison with variants in the SoySNP50K array

The two VCF files were merged on common SNP positions using BCFtools^39,40^. Heterozygous SNPs were treated as missing data before creating a similarity matrix. Within the merged VCF file, missing data and heterozygous SNPs combined accounted for <1.5% (Figshare file F6^11^). The R package SNPRelate^41^ was used to create a similarity matrix of the merged datasets and compare the accessions to one another. The script extractTop-Match.pl, available on GitHub^42^, was used to extract the top matches for a given PI in a similarity matrix (Figshare file F7^11^).

## Data Records

The authors declare that all data reported here are fully and freely available from the date of publication. All major data products are indicated in the Data Dictionary at the National Ag Library project data collection^43^. The sequencing data have been deposited in the NCBI Sequence Read Archive projects SRP062245^44^ and SRP105183^45^. We used 376 accessions raw data (331 *G. max* and 45 *G. soja*) from the SRP105183^45^ and selected 105 accessions (98 *G. max* and 7 *G. soja*) from the SRP062245^44^ for the analysis. Variant data and accession information are available at the SoyBase^46^ project page^30^ and the associated Data Store^27^. The variant data are also accessible for interactive exploration at SoyKB^26^. Also, all data including the detailed information of the accessions, phylogenetic tree, variant data are available at the National Agricultural Library Ag Data Commons (10.15482/USDA.ADC/1518301)^43^ and at figshare^11^.

## Technical Validation

### Assessment of variant calls and accessions relative to the U.S. soybean germplasm collection

All but 5 of the 481 accessions sequenced in this study have a counterpart in the U.S. germplasm collection (sharing the same PI accession number). The VCF file was merged with the SoySNP50K VCF file from the SoyBase Data Store^47^, which contains genotype information for the entire USDA soybean germplasm collection^48^, based on common SNP positions. The R program SNPRelate^41^ was used to create a similarity matrix of all of the lines in the VCF file. In soybean, there is known variation between accessions with the same name^49^, however, it is expected that the top match for each of the 481 accessions would be the accession with the same PI name from the SoySNP50K dataset. The similarity scores between the re-sequenced accessions and its SoySNP50K counterpart ranged from 99–55%, with an average similarity score of 0.987. Of the 481 accessions, 441 had a 99% or greater similarity to their expected match, while 20 lines had a similarity between 90–99% to their expected match (Figshare file F5^11^). Of the 461 accessions that had a similarity >90% to their counterpart, 407 accessions had the same PI identifier as the top match from the SoySNP50K. In 54 cases, the top hit from the SoySNP50K was not the expected PI accession number, but the difference in percent identity between the expected accession and top match was within 3%. These results indicate genetic redundancy for some groups of genotypes in the USDA soybean germplasm collection.

The 5 accessions that did not have a counterpart in the SoySNP50K dataset had similarity <90% to all other lines, indicating that these lines are unique to this study. Fifteen accessions were labeled as mis-assigned, as these accessions had a >3% similarity difference between the expected top match and what was observed, indicating an error in the accession’s identity. These differences could be due to the fact that some accessions were collected at markets that included a mixture of seed. This explanation may apply for PIs PI 407262, PI 424079, PI 437160, PI 639586, PI 360957, PI 628913, PI 639559B and their respective top matches: PI 407264, PI 424048, PI 437153 A, PI 639610, PI 379563, PI 628917, PI 639558. For all of these cases, the PIs and their top matches all have the same country of origin. For example, PI 407262 has common name K42-A and is from South Korea, its top match is PI 407264, also from South Korea, and with a similar common name of K42-C. The top match of accession PI 548402 is to PI 438497. These accessions have the same common name, ‘Peking’, which could have led to a mis-labeling, as there are 7 lines in the USDA Germplasm Resources Information Network (GRIN) with the common name ‘Peking’. This also illustrates that two accessions with the same common name may not be genetically identical – or even particularly similar. Considering accessions that had a 90-99% similarity to their U.S. counterpart, we found that in the SoySNP50K data, there are two accessions that reference soybean line Hutcheson (‘Hutcheson’ and ‘PI 518664’). There are differences between these lines due to ‘Hutcheson” being grown out for multiple generations in a lab after seed was obtained from GRIN, while PI 518664 was sourced directly from GRIN (Qijian Song, personal communication.). We suspect that a 90-99% similarity score to the SoySNP50K counterpart could be due to this event when 2 accessions have been grown out separately for multiple generations and inadvertent selection occurs.

### Genomic diversity

Resequencing of 376 accessions yielded 680 billion 125-bp paired-end reads, resulting in 6.5Tb of high-quality raw data (Figshare file F3^11^). This 6.5 Tb data was combined with 1 Tb (105 selected accessions) of publicly available raw data^3^. Sequence reads were aligned to soybean reference genome Wm82.a2 (downloaded from Phytozome), using the BWA aligner^21^. The mapping rate varied from 97.52%-99.50%, averaging 99.14% in the *G. max* lines and 97% in *G. soja* (Figshare file F3^11^). From the mapped sequence a total of 7,869,806 SNPs were identified.

### Variant effects

The snpEff program^28^, used to predict SNP effects, labeled 7,590,330 SNPs as “modifier” (falling in intergenic regions), 125,602 as having a “low” effect (causing a synonymous mutation), 146,236 as having a “moderate” effect (causing a non-disruptive change in the protein), and 7,638 as having a “high” effect (causing a disruptive change in the protein). The 7,638 SNPs predicted as having a “high” effect are in 5,987 genes (Figshare file F5^11^ and file glyma.Wm82.gnm2.div.G787.snpEff.gff3 at the SoyBase Data Store^27^). The SNPs, along with their snpEff annotation, can be viewed on the SoyBase genome browser (https://soybase.org/gb2/gbrowse/gmax2.0/) under the “naturally occurring sequence variants” track, as “USB481”.

SnpEff was run on the *G. max* and *G. soja* data files glyma.Wm82.gnm2.div.G787.USB481_nosoja.vcf.gz and glyma.Wm82.gnm2.div.G787.Soja.vcf.gz at the SoyBase Data Store^27^, giving the respective snpEFF files at that location. The *G. max* results indicated 4,661,844 SNPs as a “modifier”, 76,352 as having a “low” effect, 82,567 as having a “moderate” effect, and 4,041 as having a “high” effect. The 4,041 SNPs as having a “high” effect fall into 3,420 genes. The *G. soja* results indicated 9,356,860 SNPs as a “modifier”, 187,992 as having a “low” effect, 212,196 as having a “moderate” effect), and 10,326 as having a “high” effect.

## Usage Notes

The variant data for the 481 diverse soybean accessions have many potential uses. We illustrate with four analyses: calculation of linkage disequilibrium decay; visualization of regional conservation and difference between accessions; and phylogenetic and structural analyses. Online tools for visualizing similarities and differences for the 481-accession data set are described below. The phylogenetic analysis and associated files are also available for interactive exploration.

### Linkage disequilibrium analysis

A set of 25,495 SNPs in-common between the resequencing results and the SoySNP50K haplotypes^48^ were used to survey the rate of LD decay and population structure in the collection. The LD for each chromosome and the average LD across all 20 chromosomes is indicated in Table 1. The average LD decay distance value for the 429 *G. max* accessions is 173 kb, at *r*^*2*^ = 0.2. (The 45 accessions of *G. soja* were too few to calculate LD decay that is meaningfully comparable to LD for the *G. soja* accessions). There is no clear correlation (*r*^*2*^ = 0.33) between chromosome size and LD decay distance (Table 1, Supplementary Figure 1). The LD decays at approximately the same distance (0.1 Mb) in both the shortest and second longest chromosomes (chromosomes 11 and 1).

### Conservation and divergence of genomic regions

The tool GCViT (Genotype Comparison Visualization Tool)^37,38^ available at SoyBase (https://soybase.org/gcvit/) was used to identify genomic regions that differ between accessions. A small subset of cultivated and wild accessions were selected randomly to compare against the elite cultivar Essex (PI 548677) (Fig. 3). The three *G. soja* accessions were selected to include one from each county of China, Japan, and Korea. Essex was chosen to be used as a reference because of its importance in soybean breeding programs. Five of the 20 chromosomes are displayed in the figure. The heatmaps show blocks of conserved regions between cultivated accessions on chromosomes 5 and 20. Similar results are produced when comparing any cultivated soybean to wild within this dataset. Darker regions correspond to genomic regions that differ greatly between Essex and the accession of comparison whereas the lighter regions show shared genomic regions. Conserved regions could indicate genomic regions that were selected during domestication, as similar results (e.g. conservation on chromosomes 5 and 20) were reported by Han *et al*.^50^. Also noteworthy is a region in the middle of chromosome 15, where Essex shares a genomic region with accessions PI 209332, PI 548359 (Kingwa), and PI 567357, but is different between all other accessions, suggesting selective introgressions. Highly similar results are produced using different elite cultivars as a reference and comparing other landrace and wild cultivars. For example, if Lee (PI 548656) is used as the reference instead of Essex, we see the exact same results on Gm05 and Gm20. Results can be tested/confirmed/explored using the GCViT tool^37,38^ at SoyBase and selecting the USB481 dataset.

### Phylogenetic and structure analysis

25,496 SNPs from this study that were in common with the SoySNP50K iSelect BeadChip-derived data^14^ were used to develop a phylogenetic tree showing similarity relationships among accessions (Fig. 1). The tree is rooted between *G. max* and *G. soja* accessions. In both the *G. max* and *G. soja* clades, the accessions generally cluster by country of origin, as indicated by groupings of colors (countries): Japanese (green), Korean (orange), Chinese (blue), U.S. (light blue), and all other countries in gray. This suggests that phylogenetically distinct lineages arose within particular geographic locations, with relatively limited genetic exchange between, for example, central China and Japan. The accessions near the top (Fig. 1, predominantly dark blue clade, extending from PI 567357 and ending prior to Fiskeby III) are primarily from China while the U.S. elite lines are mostly in the lower middle (green clade, extending from Fiskeby III through PI 95860). A few accessions are highlighted and named in the figure, in view of their importance in U.S. breeding programs. Several cultivars near the top of the tree have been important sources of disease resistance: PI 88788 and Peking for SCN resistance, and Kingwa for Phytophthora resistance. Fiskeby III and Lee are tolerant to salt^51^, while Essex, Hutcheson, and IA3023 are common parents in many breeding programs. *G. soja*, PI 483463, is highlighted in the figure due to its genome having been recently sequenced and made available^9^.

To complement the phylogenetic analysis, a genomic Structure plot for all 481 accessions was generated based on a cluster size of K = 5 (Fig. 2). The order of the accessions in the Structure plot is the same as in the phylogenetic tree (Fig. 1). The Structure results generally correspond with the phylogenetic results – for example, with *G. soja* lines (yellow) occur together, as expected considering their genetic distance from *G. max*.

In this study, we have presented re-sequencing data of 481 diverse accessions, including 52 wildtypes (*G. soja*). 7.8 million SNPs have been identified and more than 5,900 genes with high effect changes have been discovered among the germplasm collection. These changes will be of use in soybean breeding programs.

Among the USDA soybean germplasm collection, we have identified 15 accessions that are potentially misnamed which will help other researchers avoid errors in their analyses. Illustration of the data with phylogenetic and structure analyses highlights the history of soybean domestication through mostly independent selection in numerous locations across Asia.

The results presented here help build a more complete history of the US soybean breeding programs, which in turn will guide future efforts in soybean breeding.

## Supplementary information

Supplementary Figure 1

## Data Availability

Scripts used to extract information from similarity matrix can be found on Github^42^.
