# Supplementary material for: Genetic variation among 481 diverse soybean accessions, inferred from genomic re-sequencing
Source: Sci Data. 2021 Feb 8;8:50. doi: 10.1038/s41597-021-00834-w (PMC7870887; doi:10.1038/s41597-021-00834-w)
Supplement: Supplementary file 1 — Supplementary Figure 1 [file 41597_2021_834_MOESM1_ESM.pdf]

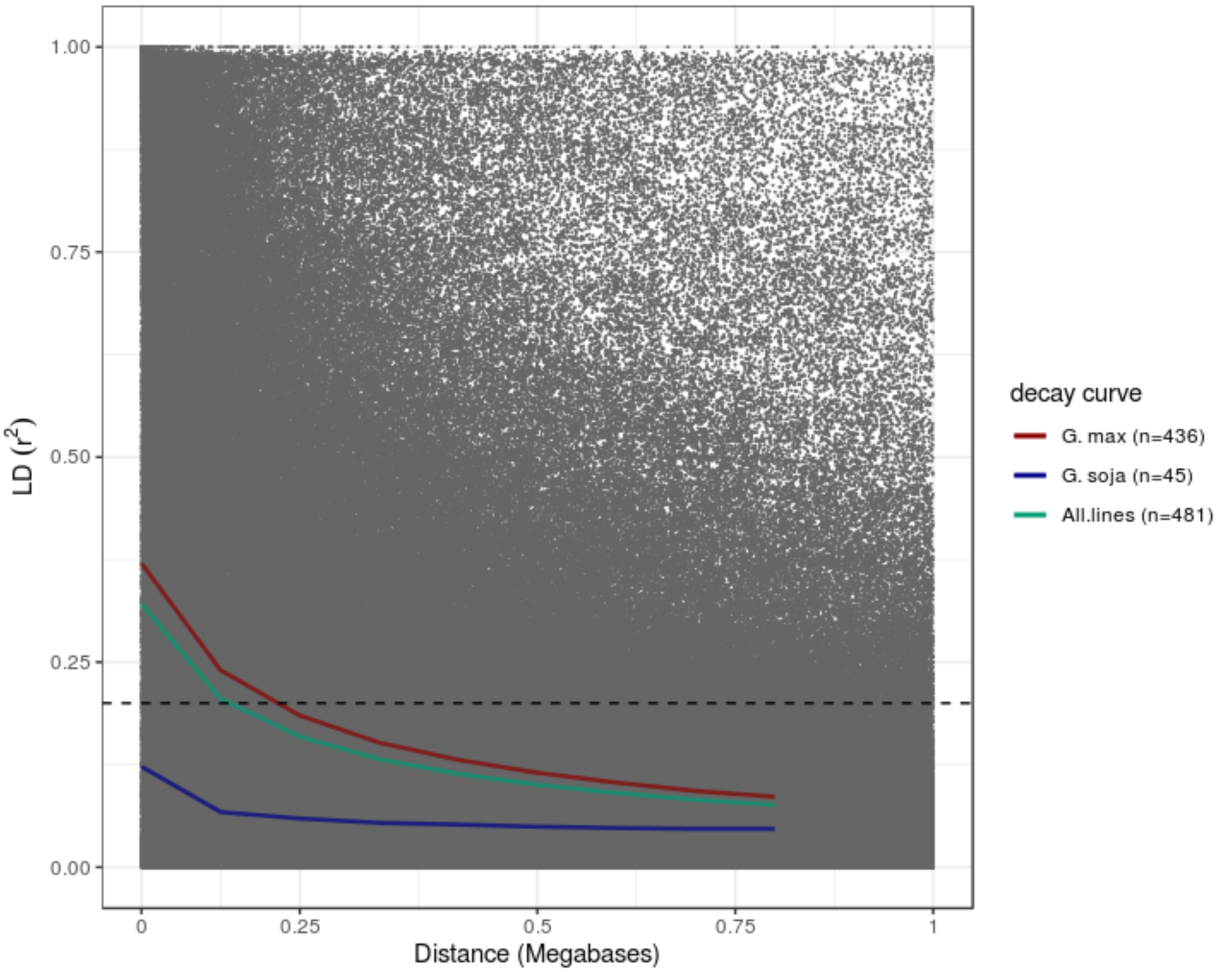

Supplementary Figure 1. Average LD decay (r^2) as a factor of physical distance (Mb) across all 20 soybean chromosomes. The decay curve for all the accessions is indicated by a green line while decay in G.max accessions is indicated in red, and the decay in G.soja accessions is indicated in blue.
